# Microstructured Block Copolymer Surfaces for Control of Microbe Adhesion and Aggregation

**DOI:** 10.3390/bios4010063

**Published:** 2014-03-14

**Authors:** Ryan R. Hansen, Katherine R. Shubert, Jennifer L. Morrell-Falvey, Bradley S. Lokitz, Mitchel J. Doktycz, Scott T. Retterer

**Affiliations:** 1 Center for Nanophase Materials Sciences, Oak Ridge National Laboratory, Oak Ridge, TN 37831, USA; E-Mails: hansenrr@ornl.gov (R.R.H.); lokitzbs@ornl.gov (B.S.L.); doktyczmj@ornl.gov (M.J.D.); 2 Biosciences Division, Oak Ridge National Laboratory, Oak Ridge, TN 37831, USA; E-Mails: krshubert@gmail.com (K.R.S.); morrelljl1@ornl.gov (J.L.M.-F.)

**Keywords:** lectins, exopolysaccharide, affinity-based capture, cell adhesion, cell aggregation, block copolymers, biofilms

## Abstract

The attachment and arrangement of microbes onto a substrate is influenced by both the biochemical and physical surface properties. In this report, we develop lectin-functionalized substrates containing patterned, three-dimensional polymeric structures of varied shapes and densities and use these to investigate the effects of topology and spatial confinement on lectin-mediated microbe immobilization. Films of poly(glycidyl methacrylate)-*block*-4,4-dimethyl-2-vinylazlactone (PGMA-*b*-PVDMA) were patterned on silicon surfaces into line arrays or square grid patterns with 5 μm wide features and varied pitch. The patterned films had three-dimensional geometries with 900 nm film thickness. After surface functionalization with wheat germ agglutinin, the size of *Pseudomonas fluorescens* aggregates immobilized was dependent on the pattern dimensions. Films patterned as parallel lines or square grids with a pitch of 10 μm or less led to the immobilization of individual microbes with minimal formation of aggregates. Both geometries allowed for incremental increases in aggregate size distribution with each increase in pitch. These engineered surfaces combine spatial confinement with affinity-based capture to control the extent of microbe adhesion and aggregation, and can also be used as a platform to investigate intercellular interactions and biofilm formation in microbial populations of controlled sizes.

## 1. Introduction

Affinity-based capture surfaces that implement lectins as recognition elements are capable of isolating and concentrating microbe targets from complex solutions for microbe identification and profiling of exopolysaccharide content [1,2,3,4]. Lectins offer an inexpensive, non-destructive, and reversible approach to cell capture, but are often limited in capture efficiency due to their inherently weak binding affinity with complementary glycans (K_D_ ~ 10–100 μM) [5,6]. These limitations have recently been addressed through synthetic efforts focused on developing materials with clustered oligosaccharides to achieve multivalent, high-avidity binding [7,8]. Other reports have demonstrated that clustering lectins together on surfaces also improves lectin avidity towards complementary glycoproteins, thereby improving detection sensitivities [9]. Using this methodology, we recently developed cell capture surfaces containing three-dimensional, circular films of a block copolymer, poly(glycidyl methacrylate)-*block*-4,4-dimethyl-2-vinylazlactone) (PGMA-*b*-PVDMA). This polymer clustered large quantities of lectin together and promoted the capture of microbes from solution [10]. 

In addition to the biochemical surface composition, surface topological features are a second critical aspect affecting the attachment of microbes. In recent years, engineered surfaces containing periodic micro and nano-structures have been used to control or inhibit the association of microbes to surfaces [11]. For example, surfaces containing periodic, micron-scale patterns mimicking shark skin greatly reduce microbial colonization and growth and are now patterned into commercially produced adhesive films for use in healthcare and cleanroom settings [12]. At the nano-scale, post arrays have been used to manipulate the spatial organization of both gram-negative and gram-positive bacteria, thereby disrupting intercellular interactions that typically occur during biofilm formation [13,14]. Additionally, micro-scale confinement has been shown to inhibit biofilm formation in flow-based systems [15]. 

Motivated from these findings, we further develop our cell capture surfaces to contain patterned, three-dimensional PGMA-*b*-PVDMA films of varied shapes and densities in order to investigate the effect of surface topology and spatial confinement on lectin-mediated microbe attachment. Our results indicate that this material can be patterned onto surfaces in a manner that favors the adhesion of individual microbes, and that these patterns can be further manipulated to control the extent of aggregation within the adherent microbe population. Such control is critical to maintaining surfaces that can capture and release individual microbes while avoiding excessive biofouling and is also pertinent for applications requiring subsequent gene expression analysis of the isolates. This control is unachievable using conventional, two dimensional lectin surfaces and represents a potential advancement in lectin-based microbe capture.

## 2. Experimental Section

### 2.1. Materials

Poly((glycidyl methacrylate)-block-4,4-dimethyl-2-vinylazlactone), herein referred to as PGMA-*b*-PVDMA, of GMA and VDMA block lengths of 56 and 178, respectively, was synthesized and characterization as detailed in our previous report [16]. All PGMA-*b*-PVDMA was stored dried at 4 °C until use. Four-inch silicon wafers obtained from Silicon Quest were used as substrates. Triticum vulgare lectin (Wheat germ agglutinin, WGA) was purchased from Sigma, re-suspended in 1× phosphate buffered saline (PBS; 137 mM NaCl, 2.7 mM KCl, 10 mM Na_2_HPO_4_, 2 mM KH_2_PO_4_, pH 7.4) to 1 mg/mL, flash frozen with liquid nitrogen and stored at –80 °C until use. Fluorescent microbe labeling was done with fluorescent dye FM^®^ 1-43, FM^®^ 4-64, or SYTO^®^ 85 (Life Technologies). FM^®^ 1-43/FM^®^ 4-64 dye was suspended to a concentration of 1 mg/mL in 1× PBS and stored at –80 °C until use. SYTO^®^ 85 dye was stored in 5 mM in DMSO at −20 °C until use. All dyes were able to label microbes with high levels of fluorescence intensity. *Pseudomonas fluorescens* GM30 was used as the target microbe and stored in glycerol stocks at –80 °C until use [17,18]. All other chemicals were purchased from Sigma and used as received.

### 2.2. Lithographic Patterning of PGMA-b-PVDMA Films

**Figure A1:**
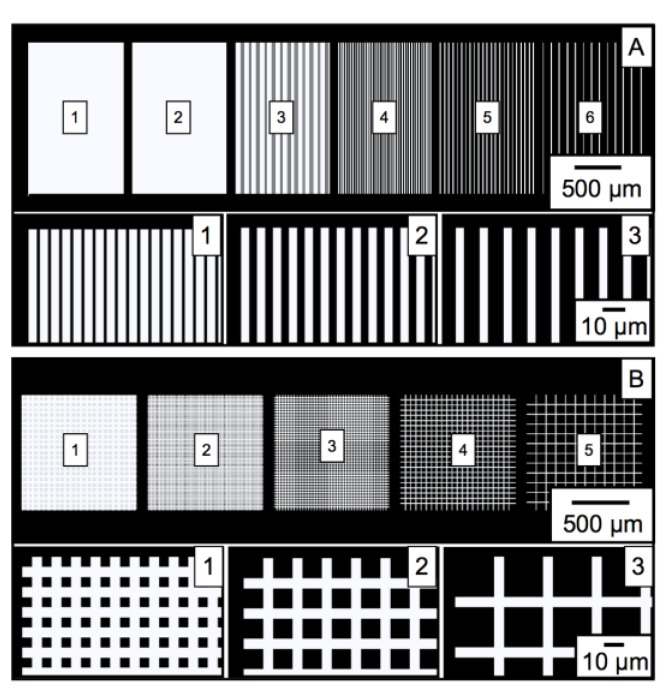
Layout of a line and grid patterns containing PGMA-*b*-PVDMA patterned films (white) on silicon substrates (black). (**A**) Top Row: Layout of the six line arrays. Line arrays have increased pitch moving from left to right. Bottom Row: Zoomed-in layout of arrays 1, 2, and 3, which contain 5 μm wide lines with a pitch of 7, 10, and 15 μm, respectively. (**B**) Top Row: Layout of the five square grid patterns. Grids have increased pitch moving from left to right. Bottom Row: Zoomed-in layout of square grids 1, 2, and 3, which contain 5 μm wide lines with a pitch of 10,15, and 25 μm, respectively.

Each wafer consisted of nine replicate pattern sets. The layout of a pattern set is described in Figure A1 (Appendix). Each pattern set consisted of six line arrays and five square grids. Within each line array was 5 μm-wide parallel rectangular strips with constant pitch (line center to line center distance). The six line arrays contained a pitch of 7, 10, 15, 25, 45 and 85 μm. Square grid patterns consisted of 5 μm-wide parallel rectangular strips running perpendicular to a second set of 5 μm-wide parallel rectangular strips. The five square grid patterns had a varied pitch of 10, 15, 25, 45, and 85 μm. The overall area of each line array was 1.6 mm^2^ and the overall area of each grid was 1.0 mm^2^. Conventional photolithographic methods were used to first modify the silicon wafers with patterned photoresist as previously detailed [10]. After photoresist patterning, substrates were treated with O_2_ plasma for 3 min, and then replicate pattern sets were diced into 20 × 20 mm substrates. Each substrate was spin coated (1,500 rpm, 15 s) with 100 μL quantities of a 1 wt% solution of PGMA-*b*-PVDMA in anhydrous chloroform and annealed for 18 h at 110 °C. Substrates were then sonicated in acetone for 5 min to remove the photoresist, leaving behind the patterned PGMA-*b*-PVDMA films. Substrates were finally rinsed in isopropyl alcohol, dried in N_2_, and stored in a vacuum desiccator until use.

### 2.3. WGA Functionalization and Microbial Incubation

*P. fluorescens* GM30 was used as a model microbe in this system and WGA lectin was used as a complementary capture protein due to the high levels of association noted in previous solution-phase binding assays and in affinity-based capture studies [10]. WGA was incubated over the patterned surfaces at 1 mg/mL for 1 h in a humid environment, then washed in a 0.05% tween 20 solution in 1× PBS to remove unbound lectin, then stored in 1× PBS until further use. As a negative control to test for nonspecific interactions between microbes and the patterned substrate, surfaces were also functionalized with BSA at the same conditions. *P. fluorescens* was cultured on LB Agar plates at 29 °C for 24–48 h and stored at 4 °C for up to one week. Liquid cultures were grown by inoculating 5 mL of LB media with a single colony and growing at 29 °C with shaking (200 rpm) to the logarithmic phase (OD_600_ ~ 0.2). The cells were then harvested by centrifugation, washed in 1× PBS and re-suspended in 1× PBS to an OD_600_ = 1.0. Fluorescent dye was then added to the washed microbes (at 4 μg/mL for FM^®^ 1-43/FM^®^ 4-64; 20 μM for SYTO^®^ 85) for 1 h while shaking at 200 rpm, then washed with 1× PBS to remove unbound dye. Microbes were finally diluted to a final concentration of OD_600_ = 0.1 in 1× PBS and incubated over the WGA-functionalized substrates for 1 h. These conditions were held constant throughout the study in order to investigate the effect of surface topography on adhesion. The substrates were then washed in 0.05% tween 20 in 1X PBS to remove unbound cells and the adherent cells were finally fixed with a 2.5% glutaraldehyde solution in H_2_O and dried via aspiration according to previous protocol [10]. Microbial adhesion to WGA-functionalized substrates was tested in triplicate.

### 2.4. Instrumentation

#### 2.4.1. Brightfield and Fluorescence Microscopy

All brightfield and fluorescence images (20×, NA 0.4/100×, NA 0.95) were taken with an upright microscope (BX51, Olympus) using a 16-bit CCD camera (Luminera Corporation, Ottowa, ON, Canada) and Infinity Capture software. All fluorescence images were taken either at 20× magnification (4 × 4 binning, 0.5 s exposure time, 1.5 gain) or at 100X magnification (4 × 4 binning, 0.5 s exposure time, 1.0 gain). Fluorescent images showing the captured microbes were used for quantitative image analysis. Brightfield images of the microbes and PGMA*-b-*PVDMA films were for visualization only and not used in image analysis. 

#### 2.4.2. Atomic Force Microscopy (AFM)

All AFM images were taken using a Park Systems AFM operating in contact mode with NanoWorld PNP-TR B cantilevers (17 kHz, 0.08 Nm^−1^). Patterned regions were imaged at 90 × 90 μm, 20 × 20 μm, and 10 × 10 μm areas. PGMA*-b-*PVDMA film thickness, maximum edge height, and average roughness (R_a_) were analyzed using XEI Park System software. R_a_ measurements were measured from a 2 × 2 μm region of interest centered directly over the edge region or over the internal PGMA*-b-*PVDMA pattern regions (regions of maximum distance from the edges). Three replicate substrates were analyzed in all measurements. 

#### 2.4.3. Scanning Electron Microscopy (SEM)

A Carl Zeiss Merlin SEM was used to image patterned PGMA*-b-*PVDMA films. Charge compensation was used during sample imaging and no sample pretreatment was required. The SEM was operated at 1.7 kV and all images were taken at 5–6.5 KX magnification. 

### 2.5. Image Processing and Data Analysis

**Figure A2:**
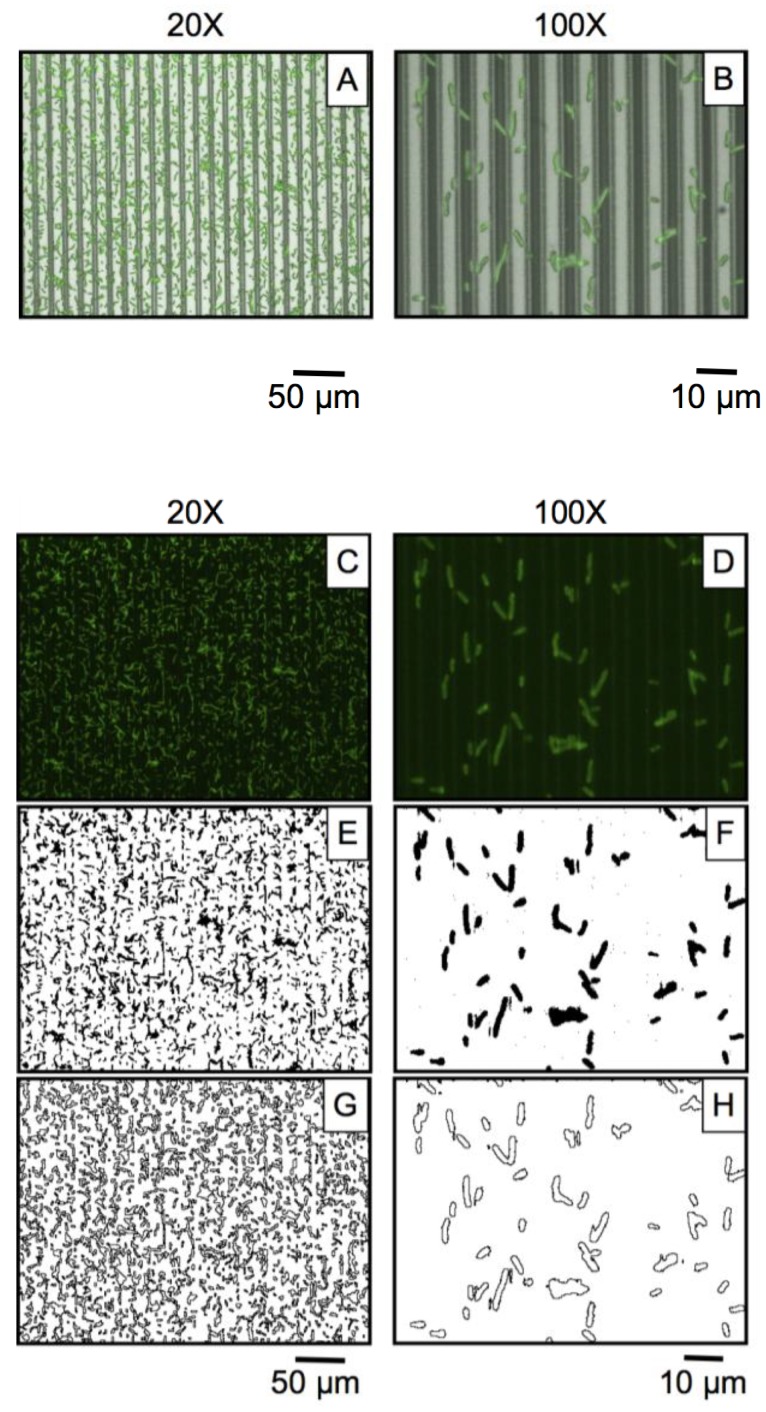
Image processing procedure for measuring microbial aggregate sizes on surfaces containing patterned PGMA-*b*-PVDMA films. (**A**) and (**B**) 20× and 100× composite brightfield-fluorescence images of *P. fluorescens* microbe captured onto WGA-functionalized PGMA-*b*-PVDMA line arrays. (**C**) and (**D**) Fluorescence images of microbes only. (**E**) and (**F**) Binary images of microbes after image processing. (**G**) and (**H**) Particle outlines for measurement of aggregate size and distribution.

Two-dimensional measurements of microbe aggregate area were determined using image thresholding and particle analysis with the Triangle method (ImageJ 1.47v). An example of the image processing method is shown in Figure A2 (Appendix). Three representative 20× fluorescent images per pattern were analyzed on each substrate and three replicate substrates were measured. This represents a total measurement area of ~2.0 mm^2^ for each pattern. Microbes both attached to the PGMA*-b-*PVDMA films and attached onto the silicon regions were included in the measurement. Histogram plots with a bin size of 10 μm^2^ were generated from this data, and all aggregates with areas >300 μm^2^ were combined into one bin. 

## 3. Results and Discussion

The purpose of this study is to investigate the impact of surface topography and spatial confinement on the lectin-mediated adhesion and aggregation of microbes. Previous studies indicated that PGMA-*b*-PVDMA was capable of forming stable, three-dimensional micropatterned films on oxidized silicon surfaces while also covalently coupling high densities of lectin. Direct comparison of lectin-mediated microbe capture between circular films of lectin functionalized PGMA-*b*-PVDMA and lectin functionalized silicon posts with similar geometries showed that the polymer enhaned microbe capture, specifically around the outer edges of the microstructure [10]. In light of these results, PGMA-*b*-PVDMA was chosen here to generate adhesive patterns with the desired topological features. Herein, we present the characterizations of these surfaces and describe their interaction with *P. fluorescens* microbes. 

### 3.1. Characterization of PGMA-b-PVDMA Films of Varied Shapes and Dimensions

**Figure 1:**
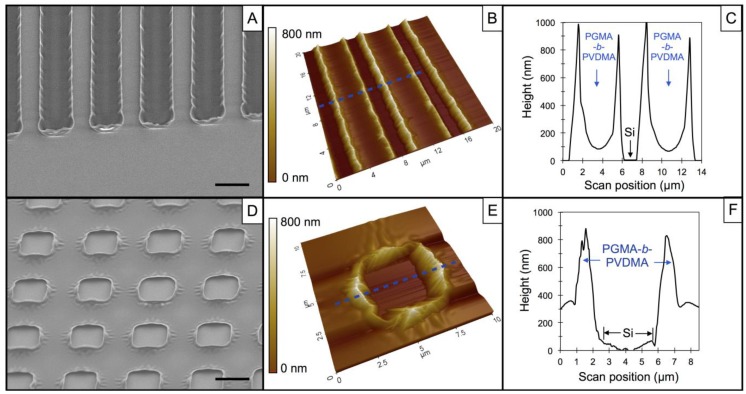
Characterization of PGMA-*b*-PVDMA patterned films. (**A**) Scanning Electron Microscopy (SEM) and (**B**) Atomic Force Microscopy (AFM) contact mode image of films patterned as 5 µm wide line arrays with a 7 µm pitch. (**C**) Cross-sectional height profile of blue line in (**B**). (**D**) SEM image and (**E**) AFM contact mode image of films patterned as square grids with a 10 µm pitch. (**F**) Cross-sectional height profile of blue line in (**E**). The scale bar in the SEM images is 5 μm.

The formation of patterned PGMA*-b-*PVDMA films was achieved through lithographic patterning followed by spin coating, annealing at 110 °C, and wet liftoff to remove photoresist. The annealing step allowed for surface attachment to occur between GMA epoxy groups and surface hydroxyl groups and also for the crosslinking of GMA groups throughout the polymer, resulting in mechanically stable films. Figure 1A–C show SEM and AFM images of films patterned as 5 μm wide line arrays with a 7 μm pitch. As shown in Figure 1(C), these films have a non-uniform cross-sectional thickness with a maximum thickness of 930 ± 32 nm occurring at the film edges. This is due to the fact that the polymer covers the ~1 μm high photoresist edges during the spin coating step. A minimal film thickness of 110 ± 20 nm occurs near the center of the film cross section. This thickness approaches the brush length for this copolymer [16]. Comparable topologies were previously noted with 10 μm diameter circular films of this material [10]. 

Films patterned as square grids with a 10 μm pitch are shown in Figure 1D–F and have similar surface topology as the line arrays. A maximum thickness of 860 ± 80 nm is found at the film edges and a minimal film thickness of 360 ± 73 nm was measured. For line array and grid films, the average surface roughness (R_a_) from the interior PGMA*-b-*PVDMA pattern region was 12 ± 2 nm and 9 ± 4 nm, respectively. In contrast, the outer edge regions of the films contain pronounced surface topography and higher average surface roughness (R_a_ ~ 180 nm for both patterns). These outer-edge features have been shown to be highly favorable for the capture of microbes after lectin functionalization [10]. The thick, pronounced edge features also serve as physical barriers that separate PGMA*-b-*PVDMA regions from flat, planar silicon regions to form a spatially confined surface environment at the length scale of a microbe (1–5 μm). 

### 3.2. Microbial Adhesion and Aggregation on WGA-Functionalized Line Arrays

**Figure 2:**
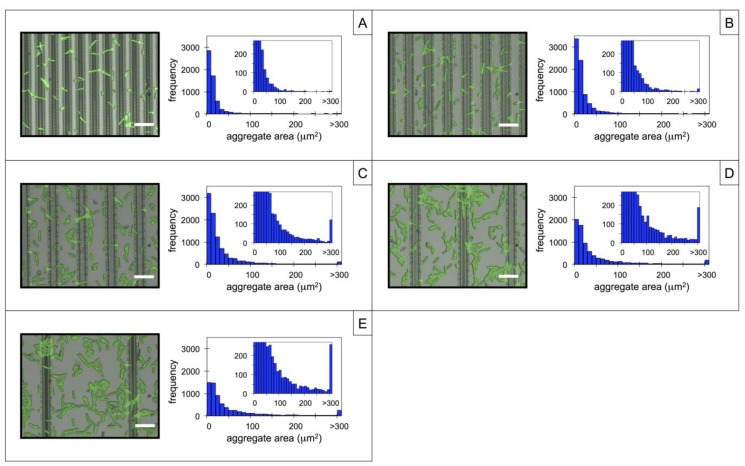
Composite brightfield-fluorescence images (100×) and corresponding histogram plots of surfaces containing PGMA-*b*-PVDMA films patterned as line arrays with a pitch of (**A**) 7 µm, (**B**) 10 µm, (**C**) 15 µm, (**D**) 25 µm, and (**E**) 45 µm after WGA functionalization and microbe incubation. Aggregate sizes were measured over a 2.0 mm^2^ area for each line array. Histogram insets are zoomed-in plots showing the frequencies of the larger aggregates. Scale bar = 10 µm in all images.

To investigate the adhesion and aggregation of *P. fluorescens* microbes over the linear PGMA-*b*-PVDMA patterned films, substrates were first functionalized with WGA capture lectins. This results in covalent immobilization of WGA onto regions of the surface containing PGMA-*b*-PVDMA films and WGA adsorption onto the silicon regions, as characterized previously [10]. After incubation with *P. fluorescens*, the microbes are immobilized over each of the patterned regions as shown in the 100× images in Figure 2A–E. In contrast, control substrates containing identical patterns but functionalized with BSA showed minimal levels of microbe adhesion (<10 cells/100× frame), which is consistent with previous reports and verifies affinity-based capture using WGA as opposed to non-specific surface adhesion and colonization [10]. 

A comparison of Figure 2A–E shows that there is a difference in the microbial aggregate size distribution that depends on the degree of spatial confinement present within the line patterns. Microbes present over the lines with the smallest pitch in Figure 2A exist predominantly as single or paired cells. This is reflected by the histogram data, showing that 79% of microbes analyzed had less than a 20 μm^2^ particle area while only 1% existed as large aggregates with areas greater than 100 μm^2^. Here, microbes appear bound across the tops and on the sides of the WGA-PGMA-*b*-PVDMA films. However, adhesion is limited by spatially restrictive access to the outer-edge regions of the films, where pronounced, microscale surface topography and high surface roughness are favorable for capture. Additionally, the topology depicted in Figure 1 causes micron-scale surface confinement that restricts the interactions between surface-associated microbes, further limiting aggregation. 

As the line pitch increases to 10, 15, and 25 μm, incrementally larger aggregates appear attached on both the WGA-PGMA-*b*-PVDMA and WGA-silicon regions of the surface, causing incremental increases in size distribution, as shown in Figure 2B–D. This shift likely occurs because as the pitch increases (pattern density decreases), the influence from planar silicon becomes greater, causing lower levels of spatial confinement at the surface. Thus, adhered microbes become more spatially available for intercellular interactions, promoting more aggregation. Little effect is noted over the last spacing step (25 to 45 μm line pitch, Figure 2D,E), where adhesion and aggregation are dominated by silicon-WGA surfaces with little crowding from the PGMA*-b-*PVDMA line patterns. Similarly, the surface coverage of immobilized microbes was also dependent on pitch. Microbe isolates that adhere onto the high-density patterns show less surface coverage than those adhered onto lower-density patterns. This is again likely due to the characteristic that flat surfaces with low levels of spatial confinement promote intercellular interaction and aggregate growth more efficiently than surfaces with high levels of spatial confinement.

These results suggest that the size of captured aggregates can be tuned based on the surface topological features and underscore the importance of highly confined features (<10 μm) for limiting microbial aggregation during capture. Similar trends with *P. fluorescens* have been reported by Diaz *et al.*, where sub-micron lines were etched into silicon surfaces and found to restrict adhesion, aggregation, and cell motility on the surface [19,20]. The ability to inhibit aggregation during microbe capture is particularly useful for assays that implement affinity-based catch-and-release of microbes for secondary genomic or proteomic analysis, which may require individual cellular isolates. For example, single cell isolation would be important to avoid changes in gene or protein expression arising from the presence of extracellular quorum sensing molecules such as acyl-homoserine lactone, which are produced at dramatically higher levels in dense populations of gram-negative bacteria [21]. 

### 3.3. Microbial Adhesion and Aggregation onto WGA-Functionalized Grids

**Figure 3:**
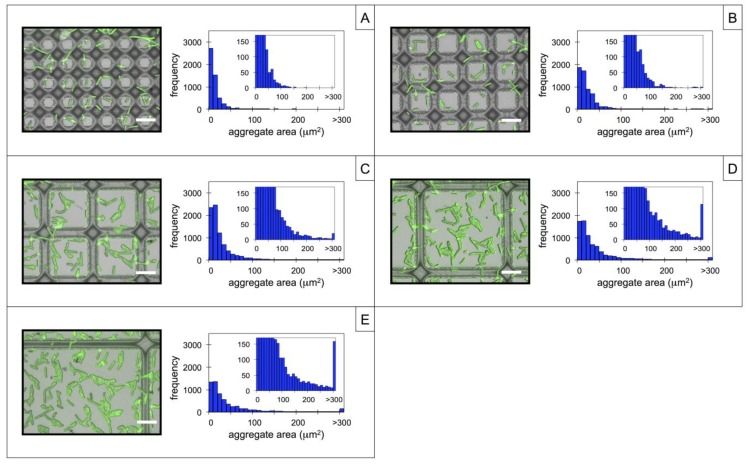
Composite brightfield-fluorescence images (100×) and corresponding histogram plots of surfaces containing PGMA-*b*-PVDMA films patterned as square grids with a pitch of (**A**) 10 µm, (**B**) 15 µm, (**C**) 25 µm, (**D**) 45 µm, and (**E**) 85 µm after wheat germ agglutinin (WGA)-functionalization and microbe incubation. Aggregate sizes were measured over a 2.0 mm^2^ area for each grid. Histogram insets are zoomed-in plots showing the frequencies of larger aggregates. Scale bar =10 µm in all images.

In an effort to further control the size distribution of captured microbes, a second dimension of spatial confinement was introduced to the patterns through the formation of square grids. Similar to the line arrays, the grid pitch was systematically varied at 10, 15, 25, 45, and 85 μm. Upon WGA functionalization and incubation of *P. fluorescens*, surface-associated microbes bound to the grids as shown in Figure 3A–E, which displays representative 100× images of each grid pattern and the respective aggregate size distributions. The general trends in microbe adhesion and aggregation noted over the line arrays in Section 3.2 were also observed over the grids. In particular, aggregate size distribution was dependent on pitch, as patterns with smaller pitch and thus higher levels of spatial confinement reduced the formation of large aggregates. In the case of the square grids with a 10 μm pitch, 80% of microbes appear as individual or pair-wise aggregates, while only 0.45% of particles had areas greater than 100 μm^2^. At larger pitches, microbe attachment again becomes dominated by the planar silicon surface, promoting higher levels aggregation.

**Figure 4:**
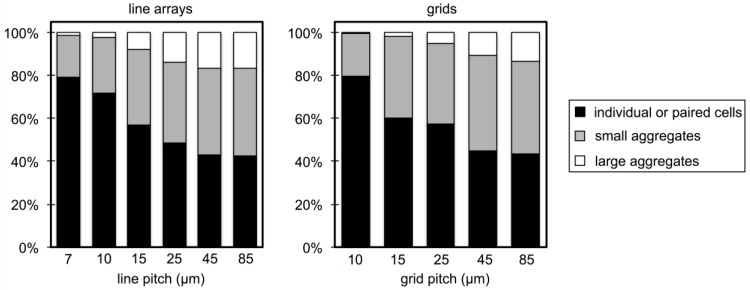
Percentage of microbes existing as individual or paired cells (<20 µm^2^), small aggregates (20–100 µm^2^), and large aggregates (>100 µm^2^) after capture and image analysis for each pitch on PGMA-*b*-PVDMA line arrays (**left**) and grids (**right**).

Figure 4 presents a direct comparison of aggregate size distributions measured between line and grid patterns. Here, immobilized microbes were binned into three size categories: (i) individual or paired cells (<20 μm^2^); (ii) small aggregates (20–100 μm^2^); and (iii) large aggregates (>100 μm^2^), and the percentage of microbes falling within each size category is displayed. For the line arrays, it is apparent that the fraction of large aggregates increases while the fraction of individual or paired cells decreases with increased pitch up to 45 μm. Grid patterns show similar trends, however the percentage of large aggregates formed over the grids is smaller compared to line arrays of the same pitch. Additionally, the influence of the PGMA*-b-*PVDMA film on aggregate size distribution is apparent within grids with a pitch up to 85 μm. These differences are likely due to the additional dimension of spatial confinement introduced by the grids, and suggest that grid geometries are preferable for the lectin-based capture of individual microbes. 

## 4. Conclusions

In this report, we have developed surfaces containing microstructured, bioactive surface polymers with varied degrees of spatial confinement to control the size of microbial aggregates that adhere through a functional binding event. While other approaches have emphasized control of microbial surface colonization by physical structures, described here is the novel combination of physical confinement and affinity-based capture. These substrates can be used for flow-based catch-and-release assays that functionally isolate microbe sub-populations as individual cells based on their exopolysaccharide content while avoiding biofilm or aggregate formation on the capture surface. As a secondary application, these lectin surfaces should also provide a route to mimicking the topology and chemical functionality of natural surfaces such as plant roots or tissues in order to gain an improved understanding of early adhesion events that occur during microbe colonization.
